# Biocompatible, stretchable and mineral PVA–gelatin–nHAP hydrogel for highly sensitive pressure sensors†

**DOI:** 10.1039/c8ra06193a

**Published:** 2018-11-01

**Authors:** Yi Zhu, Weipeng Lu, Yanchuan Guo, Yu Chen, Yuxiao Wu, Haojun Lu

**Affiliations:** Hangzhou Research Institute of Technical Institute of Physics and Chemistry, Chinese Academy of Sciences Hangzhou 310000 China luweipeng@mail.ipc.ac.cn; Key Laboratory of Photochemical Conversion and Optoelectronic Material, Technical Institute of Physics and Chemistry, Chinese Academy of Sciences Beijing 100190 China; University of Chinese Academy of Sciences Beijing 100049 China

## Abstract

Conductive hydrogels have attracted increasing attention because of their important application in flexible pressure sensors. However, designing hydrogels with a combination of excellent mechanical properties, high sensitivity, and good biocompatibility is still a profound challenge. Here we report a conductive and biocompatible PVA–Gelatin–nHAP hydrogel (PGHAP gel) by connecting a double network with inorganic nano-particles *via* ionic bonds. The as-prepared gel achieves excellent elasticity and good fatigue resistance even after 50 cycles of compression. Then a hydrogel pressure sensor was obtained using the as-prepared gel, exhibiting high pressure sensitivity almost linearly responding up to 1.5 kPa and adequate stability of the capacitance–pressure over 4 cycles. These results demonstrate the great potential applications of the hydrogel in biomedical devices, including artificial intelligence, human motion detection, and wearable devices.

## Abstract 2

A biocompatible, stretchable and mineral conductive hydrogel used for highly sensitive pressure sensors.

## Graphical abstract

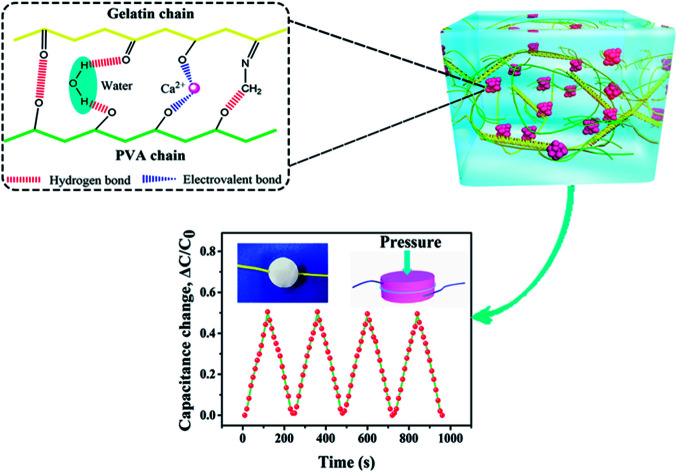

## 1. Introduction

With the arrival of the artificial intelligence era, soft and flexible pressure sensing devices are of paramount importance for a broad range of biomedical applications, such as electronic skin,^1–6^ soft robotics^7,8^ and wearable biosensors.^9–11^ Conductive hydrogels, as soft materials with a degree of flexibility similar to natural soft tissues due to their high water content, have become one of the most promising materials for pressure sensors. To date, many conductive hydrogels have been reported. Wu *et al.*^12^ developed a conductive GelMA–polyaniline (GelMA–Pani) composite hydrogel with superior electrical properties as compared to pure GelMA gel. Shin *et al.*^13^ designed a 3D HA-CA hydrogel providing dynamic, electrically conductive 3D extracellular matrix environments that were biocompatible with hNSPCs. However, this design suffered from poor mechanical strength (elastic modulus of 3.17 ± 0.56 kPa) and inferior pressure sensitivity. By chemically polymerizing with poly(3,4-ethylenedioxythiophene), Naficy *et al.*^14^ produced an electrically conductive PPEGMA/PAA/PEDOT gel with excellent mechanical strength (compressive strength as high as 11.6 MPa). But the conductivity (4.3 S cm^−1^) and biocompatibility of the gels were poor. Therefore, the profound challenge of developing conductive hydrogels with high pressure sensitivity, excellent mechanical properties and good biocompatibility still remains.

Recently, inspired by human skin, a new concept of conductive hydrogels has been put forward based on ionic transduction through hydrogels or ionic gels.^15^ A novel way of constructing double network (DN) *via* free ions diffusion has also been reported. This specific approach can effectively improve the mechanical and conductive properties of hydrogels,^16,17^ such as PEG/poly(acrylamide-*co*-acrylic acid) DN hydrogel with self-healing and electrical properties,^18^ CSH/polypyrrole hydrogels with bulk conductivity, electrical self-healing properties, and pressure sensitivity.^19^ However, their poor linear response, elasticity and biocompatibility still need to be further promoted.

Gelatin as the natural biomacromolecule constituent in cartilages, bones and skins of animals and humans, has been widely used in biomedical field^20–23^ due to its excellent biocompatibility, low antigenicity and cell proliferation.^24,25^ Herein, by introducing biocompatible PVA and mineralized nano-hydroxyapatite (nHAP), we report a bioinspired mineral PVA–Gelatin–nHAP DN hydrogel (PGHAP gel), which possesses a 3D porous skeleton with slightly dissolved free Ca^2+^ from nHAP. The PGHAP gel demonstrates good biocompatibility, fatigue resistance and outstanding conductivity. Owing to these unique properties, we design a hydrogel capacitive pressure sensor with high pressure sensitivity and stability through a facile method. In brief, this work will greatly promote the practical application of gelatin gels in the field of biological pressure sensors.

## 2. Experimental

### 2.1. Materials

Gelatin (*M*_w_ ≈ 100 kDa) was supplied from Baotou Dongbao Bio-Tech Co., Ltd. Polyvinyl alcohol (PVA 124, *M*_w_ ≈ 105 kDa) and formaldehyde solution (include polymerization inhibitors, AR) were purchased from Sinopharm Chemical Reagents Co., Ltd. Nano-hydroxyapatite (nHAP, OD ≈ 20 nm, purity 99.9%) was purchased from Beijing Dk Nano Technology Co., Ltd. Mouse osteoblasts (MC3T3-E1) were obtained from Shanghai Institutes for Biological Sciences, CAS (SIBS). Tissue Culture Plates (96 well, Corning 3599) for cytotoxicity tests and Tissue Culture Plates (Titan, SWXB-0007 and SWXB-0008) as templates were obtained from the Tansoole. Light-emitting diode (LED) bulbs (3 mm) were obtained from a store online.

### 2.2. Preparation of the PGHAP hydrogels

The PGHAP hydrogels were fabricated by a facile method. Firstly, PVA (3.33–8.33 w/v%, as part A) and gelatin (1.67–8.33 w/v%, as part B) were dissolved in ultrapure-water at 90 °C and 55 °C, respectively. Then formaldehyde (0.3 v/v%) was added into the gelatin solution as cross-linker, after being stirred for 20 min at 50 °C, the two components were mixed together, meanwhile the nano-hydroxyapatite suspension (nHAP, 2 w/v%) were uniformly dispersed into the mixture by stirring (magnetic stirring apparatus B11-2, 1200 rpm) for 30 min at 50 °C. Thus, the pre-polymer solution was prepared. Finally, the resulting pre-polymer solution was poured into template, through a 3–5 times freezing–thawing process, the PGHAP hydrogels were successfully obtained.

### 2.3. Characterization of the PGHAP gels

The mechanical properties of gels were measured by the compressive and tensile tests using an electromechanical universal testing machine (INSTRON 5966) at room temperature. For the compressive tests, cylindrical gel samples (diameter of 15 mm, and thickness of 17 mm) were compressed with a deformation rate at 5 mm min^−1^ for 80% strain. For uniaxial tensile tests, the PGHAP hydrogels with thickness of 4 mm, length of 40 mm and width of 14 mm were tested at a crosshead speed of 100 mm min^−1^. The initial Young's modulus (*E*) was calculated from the strain range of 10% to 20%. It should be noted that five specimens were tested in each group, the stress–strain curve are displayed as the one closest to the middle value, and the data are expressed as the average and standard deviation of five samples in the same group.

The chemical composition and structure features of the gels were determined by Scanning Electron Microscopy (SEM), Transmission Electron Micrograph (TEM), Thermogravimetric Analysis (TGA), Differential Scanning Calorimeter (DSC) and Fourier Transform Infrared Spectroscopy (FT-IR). For SEM observations, the samples were dried and coated with Au. Then the surface and cross section of the PGHAP gel were imaged on an Hitachi S-3500 microscope operating at 10 kV. For TEM observations, the samples were prepared by depositing the diluted pre-polymer (10 v/v%) onto a TEM grid (300 mesh), then they were dried under infrared lamp and imaged on a JEOL electron microscope at 200 kV. TGA tests were carried out in NETZSCH TG 209F1 Libra over a temperature range of 30–830 °C with a heating/cooling rates of 10k min^−1^ under N_2_ atmosphere. DSC tests were performed in NETZSCH DSC 214 over a temperature range of 0–210 °C with a heating/cooling rates of 10k min^−1^. For FT-TR measurements, the samples films were dried in an oven at 50 °C and then tested in FT-IR instrument (BRUKER TENSOR II). As parallel control, three specimens were tested in each group.

### 2.4. Fabrication of the hydrogel capacitive pressure sensor

By referring to the work of Wu' group,^5^ we designed the PGHAP hydrogel capacitive pressure sensor successfully. Initially, we prepared two round hydrogel films (diameter of 15 mm, and thickness of 3 mm) through a special template. Then, the two hydrogel films were integrated with a dielectric layer (polyethylene film, thickness of 0.12 mm) to construct a capacitive pressure sensor.

### 2.5. Measurements of conductive properties and pressure sensitivity

By using the hydrogel, a complete circuit composed of a light-emitting diode (LED) bulb was obtained. Device's ability to sense pressure changes was evaluated by using the INSTRON machine and two-probes digital multimeter. The corresponding resistance variation due to the cyclic compression was assessed while the length of the hydrogel was repeatedly changing to 25% and 50% of the original length of the specimen. In each tests three specimens were selected randomly, and the data are expressed as the one closest to the middle value.

### 2.6. Measurements of biocompatible properties

Mouse osteoblast (MC3T3-E1) cells were cultured to evaluate the cytotoxicity of the PGHAP gel. At first, the leaching liquor of the PGHAP gel was prepared by immersing the gel (1 g) in cell culture medium (10 mL) for 24 h at room temperature and filtering through a disposable needle filter (Millipore 0.22 μm). Then, 5000 MC3T3-E1 cells were dispersed in the leaching liquor (200 μL) with different concentration (0 v/v%, 25 v/v%, 50 v/v%, 100 v/v%). After that they were transferred into the cell culture plate (96-well, Corning) and cultured in CO_2_ incubator at 37 °C for 1, 3, 5, and 7 days. The cytotoxicity of the PGHAP gel was then determined through the long time dynamic living cell imaging and data analysis system (IncuCyte Zoom). In addition, the cell viability was also evaluated in terms of OD values by using a MTT assay. In order to confirm the obtained results, the gelatin gel with the same concentration of the PGHAP gel was tested in parallel as reference sample.

## 3. Results and discussion

### 3.1. Synthesis and reusability of the PGHAP hydrogel

**Fig. 1:**
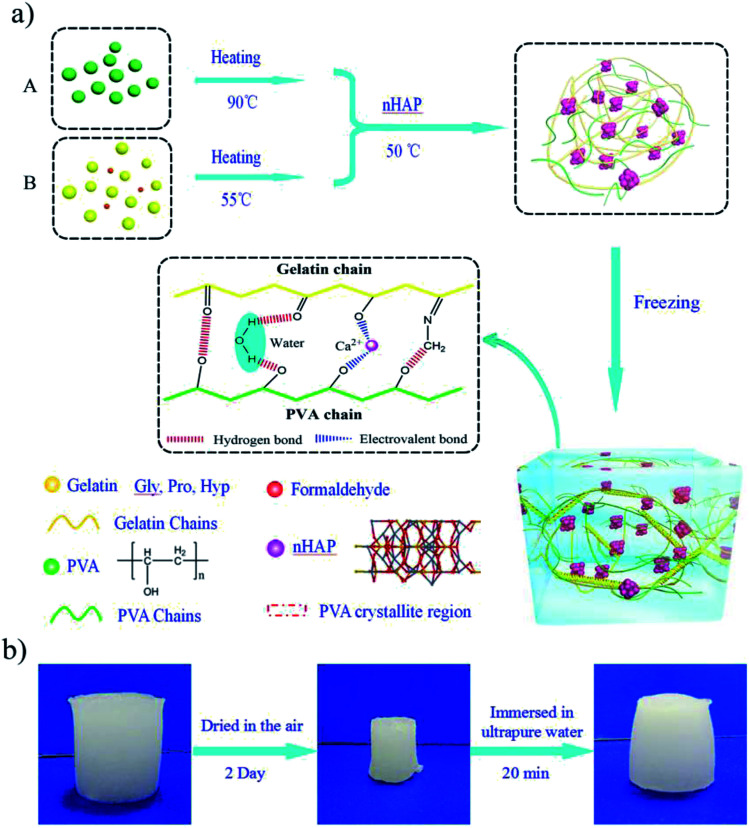
Preparation of the PVA–Gelatin–nHAP hydrogels. (a) Schematic illustration of the facial method of fabricating PGHAP hybrid hydrogels through freezing–thawing process. (b) Schematic illustration of the recoverable property of the PGHAP gel.

The PGHAP hydrogel was synthesized according to the procedure described in Fig. 1a. Formaldehyde, as cross-linker, forms covalent bonds with the amino-groups of gelatin, enhancing the strength of the composite hydrogel.^26^ Gelatin and PVA constructed a strong interpenetrating double network structure by physical cross-linking. In the pre-polymer solution, the structure of gelatin presents an amorphous state corresponded to a coil structure with primary chains. After the repeated freezing–thawing process, the crystallized state of the gelatin corresponds to the packing of triple-helixes and the coil structure stabilized by hydrogen bonds and hydrophobic interactions.^27,28^ PVA chains forms many crystallite regions, and the cross-link density greatly increases.^29^ FT-IR results (Fig. S1†) and other experiments^25,30,31^ confirm that the gelatin and PVA are bonded with each other by hydrogen bonds and hydrophobic interaction without any chemical cross-linking. Thereby, PVA physically cross-linked with gelatin and constructed a strong interpenetrating double network structure.

As an indispensable component, nHAP nano-particles were physically cross-linked with gelatin chains and PVA chains owed to the electrovalent bonding of free Ca^2+^ by the hydroxyl groups.^32,33^ This is confirmed by the FT-IR tests (Fig. S1†) which indicates that the peak corresponding to hydroxyl groups is shifting from 1086 cm^−1^ (*v*(–OH) for gelatin and PVA) to 1022 cm^−1^ and 842 cm^−1^ (*v*(–O^−^) for PGHAP). Additionally, the PGHAP gels possessed excellent electrical conductivity and reusability due to the free Ca^2+^. As depicted in Fig. 1b, when the PGHAP gel is exposed to the laboratory environment (humidity is about 20% RH) for 2 days, it shrinks gradually due to the volatilization of water. While, the dried gel can recover its original shape fleetly when immersed in ultrapure-water. Furthermore, after several times of drying–swelling process, the shape, strength and conductivity of the gel coincides with that of the original gel. These special properties mainly due to the biomineralization by the free Ca^2+^, which have also been reported by Helmut Cölfen group.^5,34–36^ Therefore, the PGHAP gel has good reusability that could reduce resources consumption. Ultimately, a biocompatible, stretchable and mineral double network PGHAP gel can be obtained.

### 3.2. Mechanical properties of the PGHAP hydrogel

**Fig. 2:**
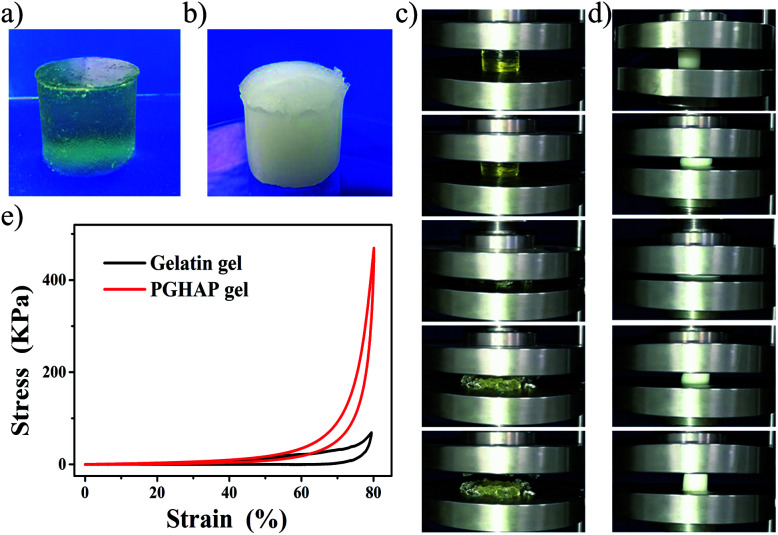
Compressive tests of the PGHAP gels. (a) The gelatin gel. (b) The PGHAP gel. (c) Photographs of the gelatin gel during a compression test. (d) Photographs of the PGHAP gel during a compression test (compressed to 80%). (e) Stress–strain curves of gelatin gel and PGHAP gel.

It is of particular importance to investigate the effects of the double network structure on the mechanical properties of the PGHAP gel. In this regard, the compressive tests of the PGHAP gel and gelatin gel were carried out. As depicted in Fig. 2, the gelatin gel (Fig. 2a) is transparent and yellowish, while the PGHAP gel (Fig. 2b) presents milky white and opaque colour. During the compressive process (compressed to 80%) of the gelatin gel and PGHAP gel (displayed in Fig. 2c and d), it is clear that the gelatin gel easily fractures under a low deformation, while the PGHAP gel can resist high stress and quickly recover its initial shape upon removing the loading force. Detailed comparisons are given by the corresponding stress–strain curves in Fig. 2e, the PGHAP gel achieves a high compression stress of 469 ± 22 kPa, which is about 6 times higher than that of gelatin gel (80 ± 5 kPa). The results indicate that the double network structure extremely enhanced the strength and stretchability of the gelatin gel.

**Fig. 3:**
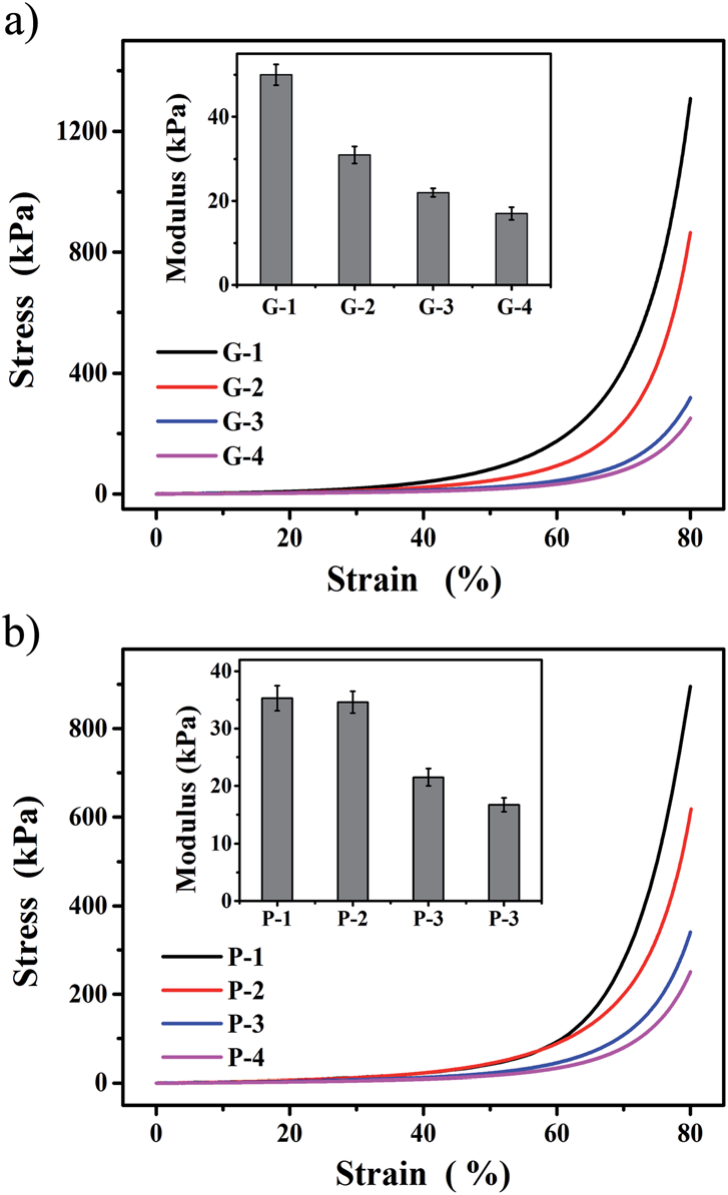
Compressive tests of the as-prepared PGHAP hydrogels. (a) Stress–strain curves and elastic modulus of the PGHAP gels with different gelatin contents (G-1n: hydrogel containing 0.25 ng g^−1^ gelatin with respect to PVA). The concentration of PVA was 0.067 g mL^−1^, nHAP was 0.02 g mL^−1^ in H_2_O. (b) Stress–strain curves and elastic modulus of the PGHAP gels with different PVA contents (P-1n: hydrogel containing 0.25 ng g^−1^ PVA with respect to gelatin). The concentration of gelatin was 0.067 g mL^−1^, nHAP was 0.02 g mL^−1^ in H_2_O.

To further gain insight into the underlying mechanism of the interaction forces on the double network and their impact on the mechanical properties of the PGHAP gel, the compressive strength of the PGHAP gels with different polymer contents was addition performed. As exhibited in Fig. 3a, the compressive strength of the PGHAP gel weakens when increasing gelatin concentration, from 1.3 ± 0.04 MPa (G-1) to 250 ± 20 kPa (G-4). The compressive modulus indicates the same tendency. In addition, the strength of the PGHAP gels with different PVA concentrations (shown in Fig. 3b) also decreased from 895 ± 32 kPa (P-1) to 250 ± 20 kPa (P-4). Usually, the mechanical strength and the elastic modulus are closely related with the concentration of the polymer. In a certain range, the increase of polymer content could effectively enhance the strength of composites in a similar manner to that obtained by improving the homogeneity of the pre-polymer solution.^37^ In our experiments, gelatin and PVA were physically cross-linked without any chemical reactions; thereby the homogeneity of the pre-polymer solution could be considered responsible for the enhanced mechanical properties of the PGHAP gel. Excessive polymer would lead to high viscosity by increasing the difficulty of mixing. In addition, high-content gelatin led to the surfeit of covalent cross-linking density, forming gel before mixing. Both factors could reduce the homogeneity of the pre-polymer solution thus decreasing the strength of the composite hydrogels.

Then detailed experiments were taken to determine the polymer concentration at a gelatin content of 0.017 g mL^−1^ and PVA of 0.067 g mL^−1^ (Fig. S2a and b). The results also confirmed that the PVA played an important role in improving the strength of the composite gels by forming the second network. Similarly, excessive nHAP also led to the heterogeneity of the pre-polymer system, and reducing the strength of the PGHAP gels (Fig. S2c), thus 0.02 g mL^−1^ nHAP was chosen. Finally, the tensile properties of the gels prepared by freezing–thawing by up to 3 (PGHAP gel-3) and 5 cycles (PGHAP gel-5) in Fig. S3,† indicate adequate tensile properties of the PGHAP gels that the tensile strength reached more than 100 kPa and the elongation at break reached 300%. Based on the above results, it could be argued that a double network PGHAP gel with excellent mechanical properties could be effectively obtained by maximizing the interaction among all components.

### 3.3. Microstructure characterization and thermal stability analysis of the PGHAP hydrogel

The microstructural characterization (SEM), the Transmission Electron Micrograph (TEM), the thermal stability analysis (TGA), and the mechanical behavior analysis (DSC) of the as-prepared gels were next examined. For comparison purposes, the corresponding experiments over gelatin gels were performed.

**Fig. 4:**
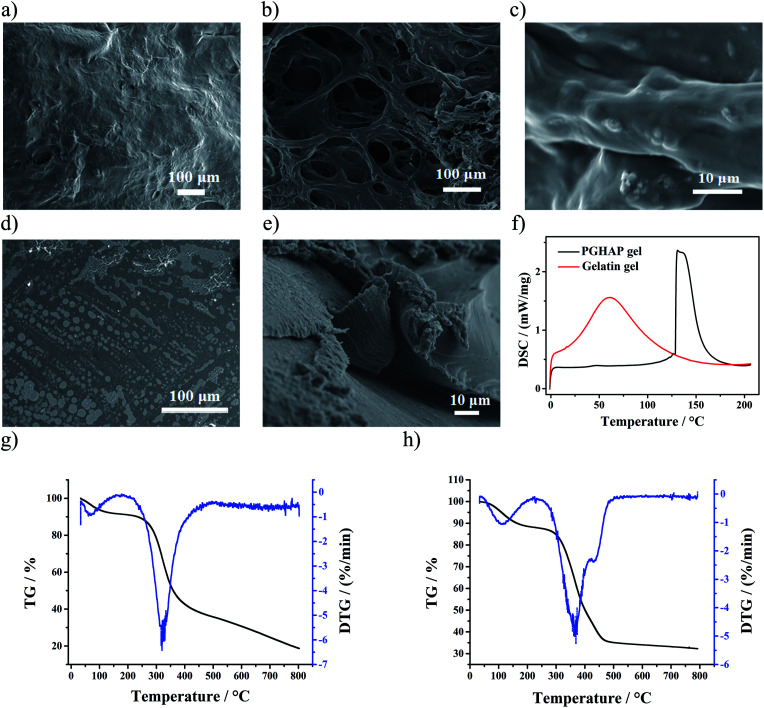
(a) SEM images of the PGHAP gel with hierarchical microscale roughness on the surface. (b) and (c) SEM images of the PGHAP gel with 3D porous skeletons. (d) SEM images of the gelatin gel with a very smooth surface. (e) SEM images of the gelatin gel with dense structure. (f) The DSC tests of the gelatin gel and PGHAP gel. (g) The TGA tests of the gelatin gel. (h) The TGA tests of the PGHAP gel.

Clearly, the SEM images of the PGHAP gel exhibits hierarchical microscale roughness (Fig. 4a), presenting 3D porous skeletons coated with nHAP nano-particles (Fig. 4b and c). However, significant morphological differences can be observed by comparing with the gelatin gel (Fig. 4d and e). The latter shows a very smooth surface with a dense internal structure without pores. Meanwhile, TEM tests show that the hydroxyapatite is in form of nano-particle and uniformly disperses in network (Fig. S4†). The results indicated that the double network offers a 3D porous structure to the composite gel, consisting it stretchable. In addition, this special structure increases ion-conducting channels of the free Ca^2+^, which effectively improved the conductivity of the PGHAP gel.

In the Fig. 4f, the corresponding DSC results are shown, demonstrating the melting processes of the two gels. Compared to the gelatin gel, the melting temperature of the PGHAP gel has been greatly improved, increasing from 62 ± 1.56 °C to 132 ± 2.35 °C. Moreover, the TGA test results (Fig. 4g and h) display that the thermal stability of the PGHAP gel has been notably improved, the thermal decomposition onset temperature increases from 275 ± 3.02 °C to 325 ± 3.24 °C. The peak at around 100 °C is attributed to the residual moisture. In short, the development of double network in PGHAP gel resulted to very stable composites as compared to the gelatin gel.

Therefore, it can be argued that gelatin chemically cross-linked with formaldehyde formed the first network, PVA chains gathered towards micro-crystalline regions consisted the second network, both interpenetrated by physical cross-linking. nHAP was uniformly dispersed in the double network structure in a form of nano-particles, physically cross-linked with gelatin and PVA *via* ionic bonds. Besides, free Ca^2+^ in the porous structure effectively enhanced the conductivity of the gel. Hence, a stable, conductive and porous PGHAP gel was successfully fabricated.

### 3.4. Design and properties of the hydrogel sensitive pressure sensor

**Fig. 5:**
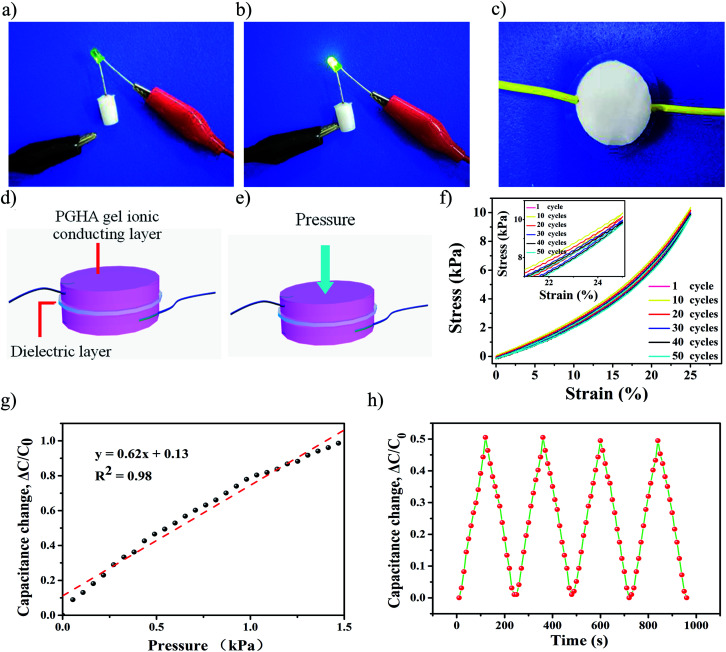
(a) and (b) A complete circuit composed of a LED bulb and our hydrogel, showing good conductivity of the hydrogel. (c) A photo of the PGHAP gel-based pressure sensor. (d) Schematic design of pressure sensor. The interfaces between the gel layers and the dielectric layer accumulate electric charge when a voltage is applied between two electrodes. (e) The schematic design for the capacitance-compression measurements. (f) Stress–strain curves of the PGHAP gels during a 50 cycles of compression process, repeatedly changing to 25% of the original length of the gel. (g) The capacitance–pressure curve of the hydrogel pressure sensor. (h) The capacitance variations due to the cyclic compression.

In order to intuitively demonstrate the good conductivity of the composite hydrogel, a complete circuit composed of a light-emitting diode (LED) bulb using the PGHAP gel was constructed. As depicted in the Fig. 5a and b, when the clip is in contact with the PGHAP gel, the entire circuit is turned on and the LED bulb lighted up, indicating its good conductivity. Furthermore, by adjusting the size of the template and the amount of pre-polymer solution, two hydrogel films were prepared. Then the two films were integrated with a dielectric layer (polyethylene film) to construct a capacitive pressure sensor (Fig. 5c). The schematic design of pressure sensor is displayed in the Fig. 5d. Due to the free Ca^2+^ of the nHAP, served as the ion carriers, the interfaces between the gel layers and the dielectric layer can accumulate electric charge when a voltage is applied between the two electrodes.^5^Fig. 5e shows the process of the capacitance-compression measurements. It is well known that capacitance devices possess high pressure and strain sensitivity except for temperature sensitivity.^38^ According to the decision formula of the parallel plate capacitor,^5,39^*C* = *εS*/4π*kd* (*C* is the capacitance, *ε* is the dielectric constant, *S* is the effective area of the conducting layer, *k* is the electrostatic constant, and *d* is the distance between the two boards), the capacitance increases with the expanding area. Therefore, when we compressed the sensor, it inevitably led to an increase in its area, thereby the capacitance increased.

Interestingly, the capacitance of the PGHAP gel pressure sensor was almost linearly responding up to 1.5 kPa. This behavior is superior as compared to that recently reported.^19,40,41^ The pressure sensitivity S was defined as the slope of the curve and it was calculated as 0.62 ± 0.06 kPa^−1^ (Fig. 5e). The durability test of the pressure sensing (Fig. 5f) reveals the stability of the capacitance–pressure over many cycles, indicating that the resistance of the PGHAP gel is ideally responded to the applied loading and unloading process within a wide stress range. Compared to various pressure sensors previously reported,^15,42^ the present one demonstrates a better sensitivity. Moreover, by using the INSTRON machine, we tested the fatigue resistance of the hydrogels after 100 cycles of compression, changing to 25% and 50% of the original length, respectively (Fig. 5e and S5†). The results show good fatigue resistance of the PGHAP gel, ensured the excellent reliability and reusability of the pressure sensor.

### 3.5. Biocompatible properties of the hydrogel capacitive pressure sensor

Because the PGHAP gel pressure sensors could be mainly employed for the detection of human health related indicators such as heartbeat, blood pressure, muscle movements, *etc.*, they inevitably come into contact with the human body. Therefore, the biocompatibility and cytotoxicity of the PGHAP gel was examined. A blank control group and a gelatin gel control group were assessed in parallel.

**Fig. 6:**
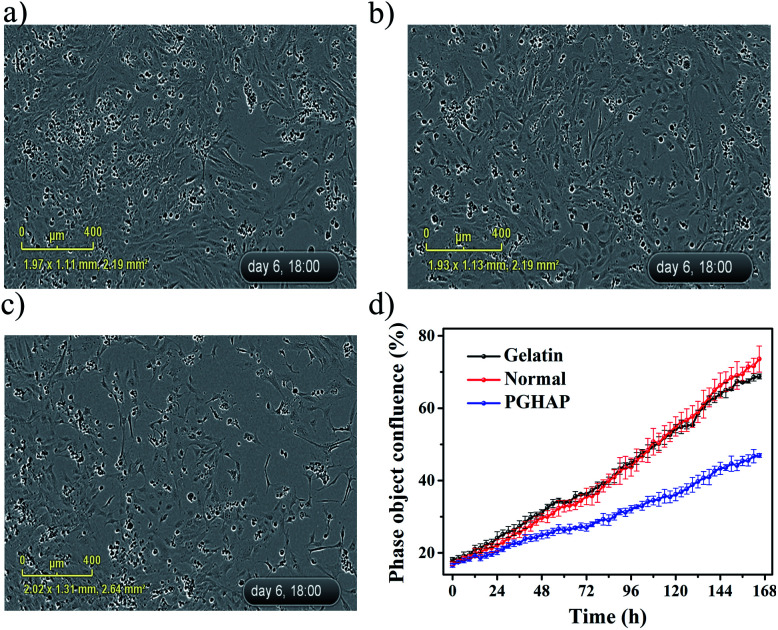
The images of the MC3T3-E1 cells cultured in the long time dynamic living cell imaging and data analysis system for 6 days. (a) The blank control group. (b) The gelatin gel control group. (c) The PGHAP gel group. (d) The results of extracts of the PGHAP gel along with control.

It can be clearly seen from Fig. 6 that the mouse osteoblasts (MC3T3-E1) grew well under the different conditions in the long time dynamic living cell imaging and data analysis system for 6 days. Compared to the control group (Fig. 6a and b), cell growth in the experimental group (Fig. 6c) was slightly inhibited. This may be due to two factors. One is the small amount of formaldehyde remained in the composite hydrogel structure that led to the death of cells. The other is the excessive polymers in the culture medium that inhibited cell growth, in accordance to previous studies^43^ and present experiments (Fig. S6†). Therefore, the slight inhibition of the cell growth in the experimental group may be extremely caused by the cooperative effect of the aforementioned factors. The statistics shown in Fig. 6d, indicate that although the growth of cells in experimental group are slightly inhibited, there is still a satisfactory growth. Moreover, the MTT tests display the alike results (Fig. S7†). Except the group of 100% cell extracts, the other groups (50% and 25% of the cell extracts) present the same trend as the control group. In a word, the PGHAP hydrogel has a good biocompatibility to be potentially applied in the field of biomedical devices.

## 4. Conclusions

In summary, we successfully fabricated and characterized of a double network PGHAP nano-composite gel with superior properties through a repeated freezing–thawing process. The PGHAP gel exhibited excellent elasticity and reusability, good conductivity and fatigue resistance after 50 cycles of compression. Based on this, a hydrogel pressure sensor was designed by a facile method which exhibited high pressure sensibility almost linearly responding up to 1.5 kPa and adequate stability of the capacitance–pressure over 4 cycles. Furthermore, the PGHAP gel showed good biocompatibility, greatly promoted their potential application in the field of biomedical devices, such as human motion detection, and wearable devices, *etc.*

## Conflicts of interest

There are no conflicts to declare.

## Supplementary Material

RA-008-C8RA06193A-s001
