# Supplementary material for: Biocompatible, stretchable and mineral PVA–gelatin–nHAP hydrogel for highly sensitive pressure sensors
Source: RSC Adv. 2018 Nov 1;8(65):36999–7007. doi: 10.1039/c8ra06193a (PMC9088965; doi:10.1039/c8ra06193a)
Supplement: RA-008-C8RA06193A-s001 [file RA-008-C8RA06193A-s001.pdf]

Electronic Supplementary Material (ESI) for RSC Advances.
This journal is © The Royal Society of Chemistry 2018

Supporting Information

Biocompatible, Stretchable and Mineral PVA-Gelatin-nHAP Hydrogel for Highly Sensitive Pressure Sensors

Yi Zhu, Weipeng Lu, Yanchuan Guo, Yu Chen, Yuxiao Wu, Haojun Lu,

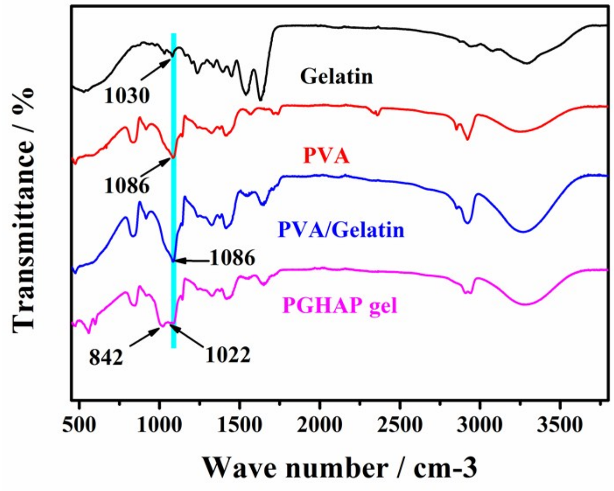

Figure S1. FT-IR spectra of gelatin, PVA, PVA/gelatin and PGHAP dry gels.

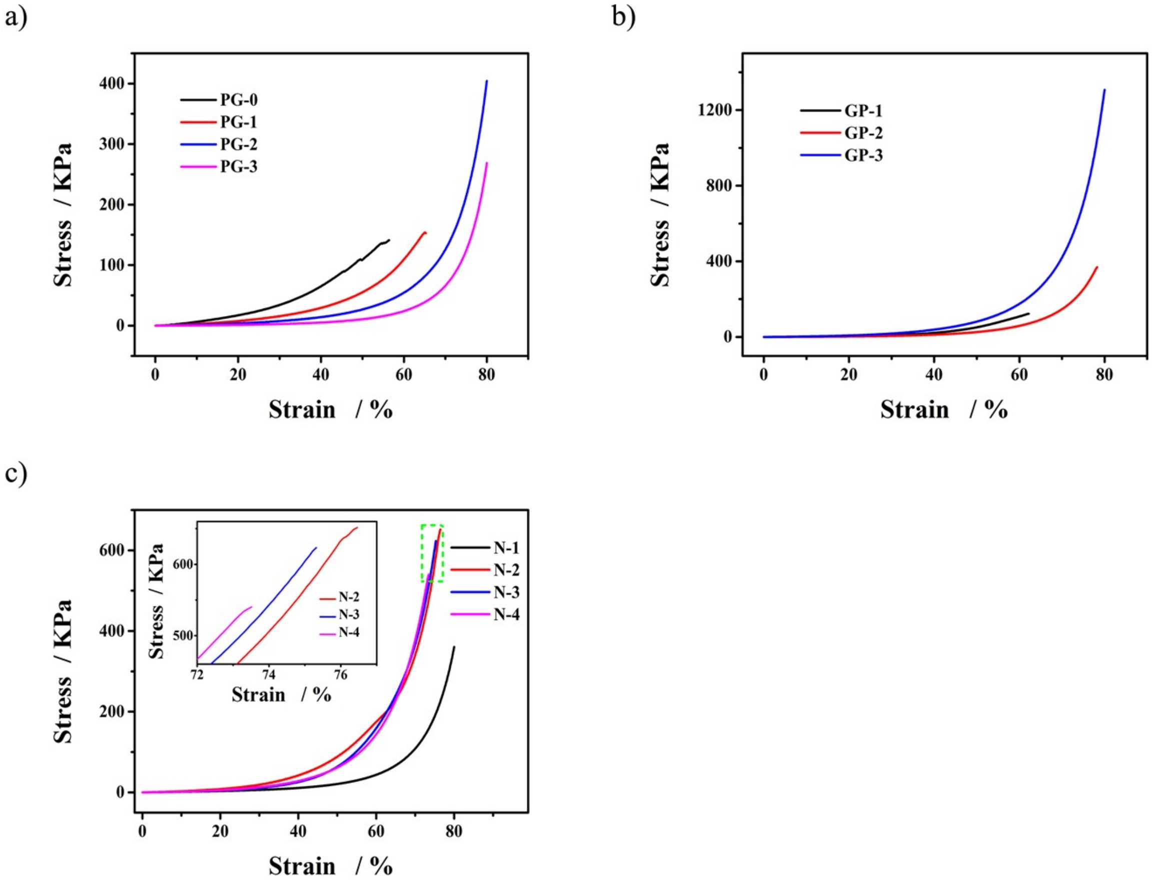

Figure S2. a) Stress-strain curves of the PGHAP gels with fixed polymer contents (0.083 g/mL). The concentration of nHAP was 0.02 g/mL in H2O, the PVA was 0 g/mL, 0.033 g/mL, 0.067 g/mL, 0.083 g/mL. b) Stress-strain curves of the PGHAP gels with different PVA contents (0.033 g/mL, 0.05 g/mL, 0.067 g/mL). The concentration of gelatin was 0.017 g/mL, nHAP was 0.02 g/mL and in H_2O. c) Stress-strain curves of the PGHAP gels with different nHAP contents(0.01~0.04 g/mL). The concentration of PVA was 0.067 g/mL, gelatin was 0.017 g/mL in H2O.

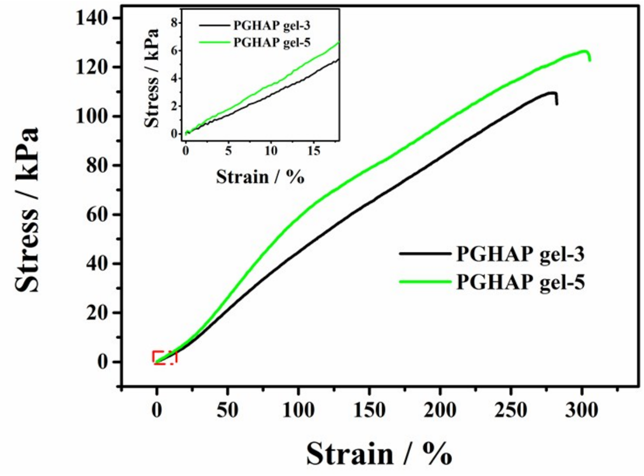

Figure S3. Stress-stain curves of the PGHAP gels during the tensil process prepared by 3 and 5 times of freezing-thawing process, respectively.

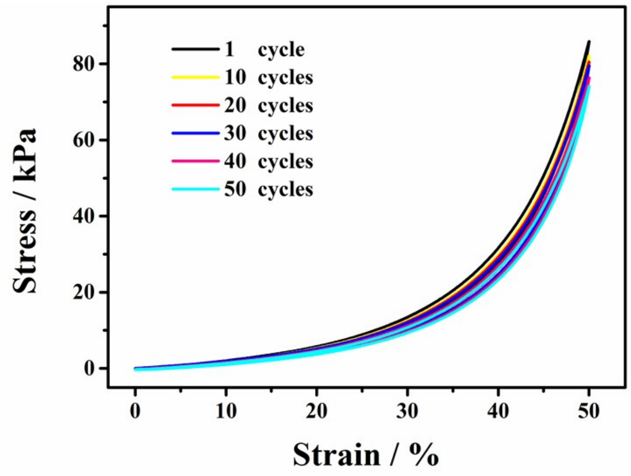

Figure S4. Stress-strain curves of the PGHAP gels during a 50 cycles of compression process, repeatedly changing to 50% of the original length of the gel.

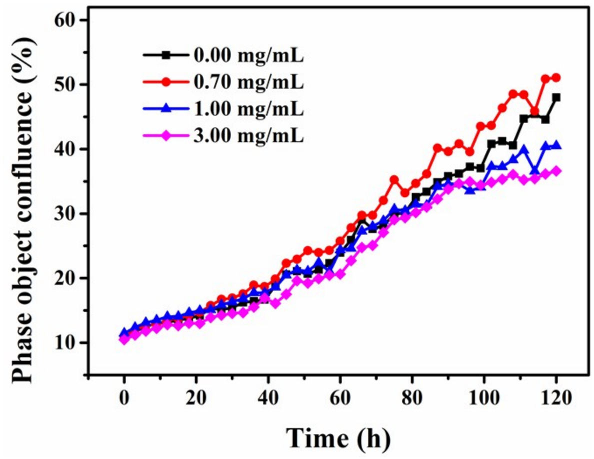

Figure S5. Effects of Different Collagen Concentration on the Growth of MC3T3-E1 cells.

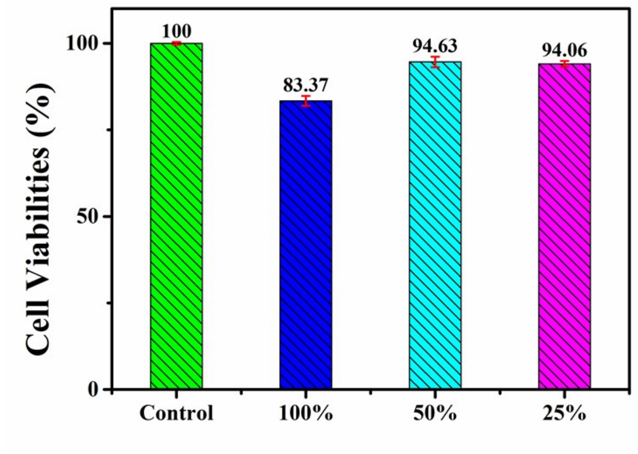

Figure S6. The cell viabilities of MC3T3-E1 cells after incubation for 72 hours with 100%, 50%, 25% extracts form the PGHAP gels.
